# Differences in gait and trunk movement between patients after ankle fracture and healthy subjects

**DOI:** 10.1186/s12938-019-0644-3

**Published:** 2019-03-19

**Authors:** Chia-Yu Hsu, Yuh-Show Tsai, Cheng-Shiang Yau, Hung-Hai Shie, Chu-Ming Wu

**Affiliations:** 1 Department of Rehabilitation Medicine, Ten-Chan General Hospital, No. 155 Yanping Rd, Zhongli Dist., Taoyuan City, 320 Taiwan; 2 Department of Biomedical Engineering, Chung Yuan Christian University, No. 200, Zhongbei Rd, Zhongli Dist., Taoyuan City, 320 Taiwan (ROC); 3 Department of Physiotherapy, Ten-Chan General Hospital, No. 155 Yanping Rd, Zhongli Dist., Taoyuan City, 320 Taiwan

**Keywords:** Ankle fracture, Trunk movement, Rehabilitation, Accelerometer, Lower extremity functional scale, Gait quality, Fall

## Abstract

**Background:**

Studies have shown that gait asymmetry and activity limitation can persist several months or years after ankle fracture. However, evidence of gait and trunk movement patterns following ankle fracture during the early rehabilitation period is scarce. Thus, we compared gait patterns and trunk movement during the early phase of rehabilitation between patients with ankle fracture and matched controls.

**Methods:**

Ten patients with ankle fractures, and ten age- and sex-matched healthy controls were prospectively enrolled. An automated infrared-assisted, trunk accelerometer-based gait analysis system was used to measure walking speed, step length, and cadence. The median time of the evaluation following ankle fracture was 4.0 months. Trunk movement intensity was evaluated as acceleration root mean square. Trunk movement symmetry and regularity were analysed using the autocorrelation method. Differences in gait characteristics between the patient and control groups were analysed using the Mann–Whitney U test. Follow-up assessment of falls was performed 24 months after the fracture. The correlations between Lower Extremity Functional Scale (LEFS) scores/falls and gait parameters were evaluated using Spearman’s rank correlation coefficient.

**Results:**

Walking speed (*p* = 0.019), step length (*p* = 0.023), cadence (*p* = 0.003), and trunk movement intensity in anterior–posterior and vertical axis (*p* = 0.001, *p* = 0.003, respectively) were all significantly lower in the ankle fracture group than in the control group. Trunk movement symmetry in vertical direction (*p* = 0.019) decreased significantly in patients with ankle fractures, whereas between-strides regularity did not differ between groups. LEFS scores were moderately correlated with walking speed (*r *= 0.60, *p* = 0.044) and step length (*r *= 0.68, *p* = 0.021). During the 24 months after the fracture, 3 falls were reported by 3 patients. Trunk acceleration root mean square ratio in mediolateral axis (*r *= 0.72, *p* = 0.018) was highly correlated with future falls.

**Conclusion:**

During early rehabilitation, patients with ankle fracture may develop trunk movement asymmetry in the vertical direction accompanied with slower walking speed and cadence, and smaller step lengths, which can contribute to muscular imbalances and potential injury. Thus, proper rehabilitation strategies should be employed for these patients.

## Background

Ankle fracture is one of the most common lower limb fractures [1]. Patients with ankle fractures often have pain, weakness, stiffness, swelling, activity limitations, and reduced participation in work and recreation [2]. Reduced participation, increased complaints of pain in the extremities and spine, and increased fatigue may affect their life in physical, psychological, and social domains years after the initial injury [3, 4]. Several studies have reported short- and long-term results after surgery [5–7]. However, most of them used radiographic tools and subjective functional evaluations to determine patient outcomes.

Evaluation of disability following ankle fracture is commonly based on indicators such as American Orthopedic Foot and Ankle Score (AOFAS) [8], Olerud–Molander Ankle Score (OMAS) [9], and Lower Extremity Functional Scale (LEFS) during rehabilitation [10, 11], which incorporate subjective or objective factors into numerical scales to describe function, alignment, and pain. Although such grading systems provide a simple way to know the degree of disability and patient’s mobility, many parameters are subjectively assessed. Furthermore, grading systems are designed to describe the general status of patients but not designed to describe subtle changes of gait parameters. Since a large majority of hospitals have limited access to a gait analysis laboratory, a more affordable, accessible, and objective method is required to quantitatively measure gait quality in a clinical setting.

Trunk accelerometry has several advantages. It is lighter, more economic and affordable than the force plates or 3-dimensional motion capturing systems [12, 13]. A tri-axial accelerometry can measure anterior-posterior (AP), Medio-lateral (ML), and vertical (VT) data of acceleration, which can evaluate the gait quality and objectively assess the rehabilitation intervention outside the laboratory [14]. It also can measure the body centre of mass movement during gait cycle [15, 16]. Reliability and validity of accelerometer-based gait analysis (AGA) systems have been established [17–20] and proved as good as a 3-dimensional motion capturing system for gait event detection and temporal-spatial parameters [21].

During normal gait cycle, trunk movement is characterized by a flexion peak near each heel strike in the sagittal plane and it reaches maximal range of motion in the frontal plane at the time of toe off [22]. Several studies have shown that trunk plays a crucial role in hemiplegic gait of stroke adults [23, 24] and crunch gait of cerebral palsy children [25]. A recent systemic review stated that stroke patients have increased, asymmetric and unstable trunk movement [26]. Moreover, the correlation between trunk position and ankle joint kinematics had been recently reported in children with cerebral palsy [25]. However, few gait or trunk kinematics studies have focused on ankle fractures in the current literature. Lower walking speed, decreased stride length and reduced internal dorsiflexion moment in the injured ankle joint following heel contact were observed in ankle fracture patients 1 year after surgical treatment [1]. Asymmetric distribution of plantar pressure following ankle fracture was noted in one study, but no perfect symmetry was found in control subjects [27]. Furthermore, surgically-treated ankle fracture patients presented limb asymmetry and decreased walking speed, measured by a portable walkway system [28]. Using an inertial measurement system at lower limb, patients with ankle fracture were found to have lower range of motion of gait cycle in thigh and calf, and a longer stride duration than the control group [29]. Wang et al. had used a 3-dimensional motion capturing system and found that patients following ankle fractures displayed deceased step and stride length; less ankle joint plantar flexion in the sagittal plane, but not in the frontal and transverse planes [30]. Although these studies found impaired temporal-spatial gait parameters and limb asymmetry in ankle fracture patients, few of them evaluated trunk kinematics (movement acceleration, symmetry and regularity) during gait cycle with an AGA system. Our primary hypothesis was that patients with ankle fracture would have lower walking speed, step length, cadence, and trunk movement symmetry and regularity than healthy controls in the first few months of rehabilitation. Understanding the characteristics of trunk kinematics and the association between gait parameters and functional measurements/future falls with impaired physical mobility in patients after ankle fractures will help clinicians develop rehabilitation plans, evaluate rehabilitation outcomes and prevent future fall events.

The primary aim of this study was to assess the gait and trunk movement differences between ankle fracture patients and able-bodied subjects with the use of a trunk AGA system in the hospital. The secondary aim was to assess the association between gait parameters and ankle functional outcomes/future falls.

## Materials and methods

### Participants

Adult participants were prospectively recruited from the rehabilitation department of a teaching hospital if they fulfilled the following inclusion criteria: ankle fracture (unimalleolar, bimalleolar, trimalleolar) treated with cast immobilization ± open reduction and internal fixation; able to walk 10 meters without a walking aid; referral to outpatient physical rehabilitation for treatment. The exclusion criteria included an injury unrelated to the ankle fracture, postoperative complications such as active infection or deep vein thrombosis, and neuromuscular conditions that could alter gait. All patients were instructed to avoid weight bearing activities for at least 6 weeks following the initial injury. Their median age was 38.0 (interquartile range: 18.0) years. Their median time of the evaluation following ankle fracture was 4.0 (interquartile range: 5.0) months. We included ten healthy individuals who were age and sex matched (± 1 years) to the ankle fracture group as the control group. Both the patients and the healthy subjects provided written informed consent. The study had been approved by the institutional review board of the hospital.

### Measuring system

**Fig. 1:**
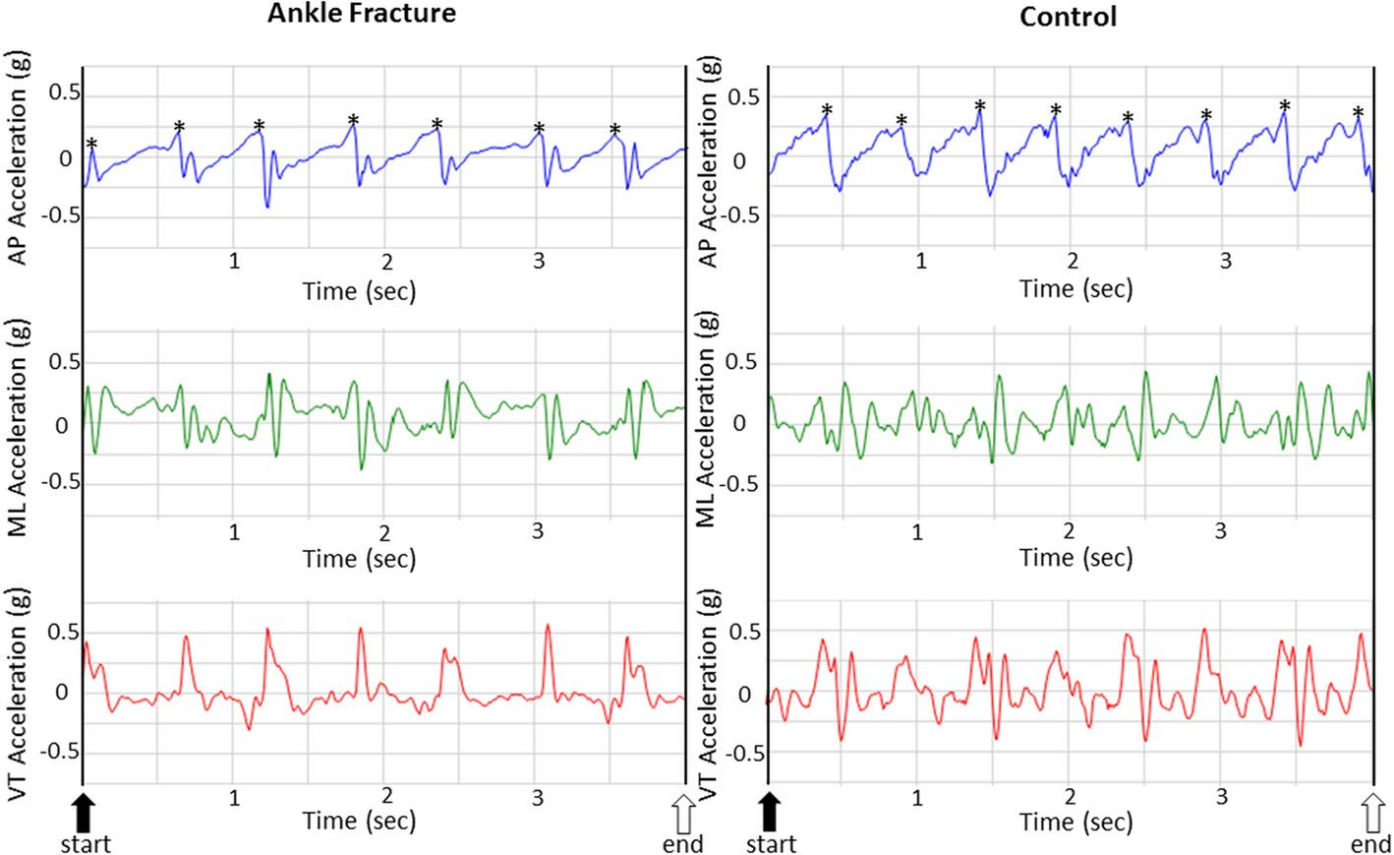
The typical plot of lower trunk tri-axial acceleration signals from both ankle fracture patients and healthy participants during 5 m of self-selected comfortable walking samples at 100 Hz. The two black bars represent the moment of infrared-defined gait initiation and termination. The black arrow indicates the start of the measurement. The white arrow indicates the end of the measurement. The asterisks around peak AP acceleration values indicates initial foot contact of each integer step. *AP* anterior–posterior, *ML* medio-lateral, *VT* vertical

The gait analysis system used in the present study was composed of a tri-axial accelerometer (ADXL345, Analog Devices, Norwood, MA, USA), an infrared unit, a laptop computer, and one strap, which had been published elsewhere in detail [20]. The tri-axial accelerometer was embedded in a wireless sensor unit, measuring 69.5 × 45.5 × 14.5 mm (length × width × height) in size. This automated infrared-assisted, trunk AGA system could reliably detect temporal-spatial gait parameters (walking speed, step length, cadence), as well as trunk movement symmetry and regularity during gait cycle in the hospital environment [20]. In brief, the accelerometer was calibrated before starting measurements. When a subject passed the infrared unit at the starting line, the system would automatically collect trunk accelerometric data. Linear accelerations were measured along the AP, ML, and VT axes, sampled at 100 Hz, and synchronized (Fig. 1). When a subject passed the infrared unit at the end line, the system would also automatically stop. The data of 5 m walk was real-time automatically analysed and stored in the gait analysis system immediately after the measurement completed [20].

### Experimental protocol

Each subject was asked to stand while the accelerometer was secured, using a strap, on the midline of low back between the L3 and L4 vertebrae. Participants were allowed walking two times for trial before the real measurement. Then, participants were asked to walk twice for a total of 8 meters, which allowed 1.5 m for gait initiation and termination, respectively. The following mean value of two measured parameters were evaluated: walking speed, step length, cadence, trunk movement symmetry and regularity, acceleration root mean square (RMS) in the AP, ML, VT directions, and acceleration RMS ratio (RMSR) in the ML direction.

### Gait parameters

*Walking speed* was calculated by dividing the 5-m walking distance by the walking time.

*Step count* was determined by the formula: *Total step count *=* Integer step count *+* the initial step *+* the final step* [20]. The integer step count was determined by the AP acceleration peak at the time of heel strike (Fig. 1) [17]. With infrared assist, the initial and last step count was estimated by the time interval between the first/last integer step and the start/end line.

*Cadence* was calculated by dividing the total step count by the walking time.

*Step length* was calculated by dividing the 5-m distance divided by the total number of steps taken.

*Acceleration RMS* in the AP, ML, VT directions was utilized to represent trunk movement intensity [31], the average magnitude of acceleration along each three-dimensional axis, during the 5-m walking period.

*Acceleration RMSR* in the ML direction represents the ratio between acceleration RMS in ML direction and the acceleration RMS vector magnitude [32]. RMSR could be useful as RMS normalization, since RMS is highly sensitive to walking speed [31]. RMSR in ML direction is a more effective measure for detecting gait differences than that in AP and VT directions because it does not correlate with preferred walking speed and was found to be a potential indictor of gait abnormality [32].

*Symmetry and Regularity* of trunk movement in AP and VT directions was estimated from the auto-correlation (AC) method proposed by Moe-Nilssen [33]. The AC coefficient is an estimate of the similarity of the time series with itself at a given time-shift. The first (Ad1) and second (Ad2) dominant period represent phase shifts equal to one step and one stride, respectively. (Between-step) symmetry of trunk movement was the value of autocorrelation coefficient corresponding to Ad1 dominant period. (Between-stride) regularity was the value of the autocorrelation coefficient at Ad2 dominant period, which expressed the similarity between strides over time. The closeness of the AC coefficient to 1.0 reflects high symmetry or stride regularity [22].

### Lower Extremity Functional Scale (LEFS)

Before the gait analysis, the ankle fracture group was evaluated for activity limitations using the Chinese version of LEFS [10]. The LEFS was designed to assess the functional status of patients with orthopaedic conditions of the lower extremity [11], such as ankle fractures [34]. The items are rated on a 5-point scale, from 0 (extreme difficulty/unable to perform activity) to 4 (no difficulty), allowing us to evaluate the degree of difficulty in doing different physical activities. The LEFS is scored between 0 and 80, with 0 indicating worst functional status and 80 indicating optimal function. This questionnaire was completed by the patients.

### Fall assessment

Patients who completed the gait analysis were contacted by telephone 24 months after the ankle fracture. The following questions were asked: Did you fall during the 24 months after the fracture? A fall was defined as the patient unintentionally coming to rest on the floor or a lower level that is not because of a major intrinsic event.

### Statistical analysis

Nonparametric 1-sample Kolmogorov–Smirnov tests were calculated to compare the observed cumulative distribution function for the continuous variables with the normal theoretical distribution. Group differences for age, body height, body weight, and sex were assessed using Fisher’s exact test or Mann–Whitney U test. Differences between the control and patient groups for gait characteristics were analysed using the Mann–Whitney U test. The Spearman’s rank correlation coefficient was used to identify associations between gait parameters and LEFS/future falls. Correlation coefficients were interpreted as follows: 0.9–1.0, very high correlation; 0.7–0.89, high correlation; 0.5–0.69, moderate correlation; 0.26–0.49, low correlation; and 0–0.25, little if any correlation [24]. Sample size calculations were based on detecting 20 cm/s [35] in the walking speed between two groups, assuming a standard deviation of 29 (cm/s), a two-tailed test, an alpha level of 0.05, and a desired power of 80%. Based on these calculations, the minimum sample size was estimated to be 6 participants per group. The data were analysed using SPSS software, version 21.0 (IBM Corp., Armonk, NY, USA). The significance level was set at *p* < 0.05 for all tests.

## Results

**Fig. 2:**
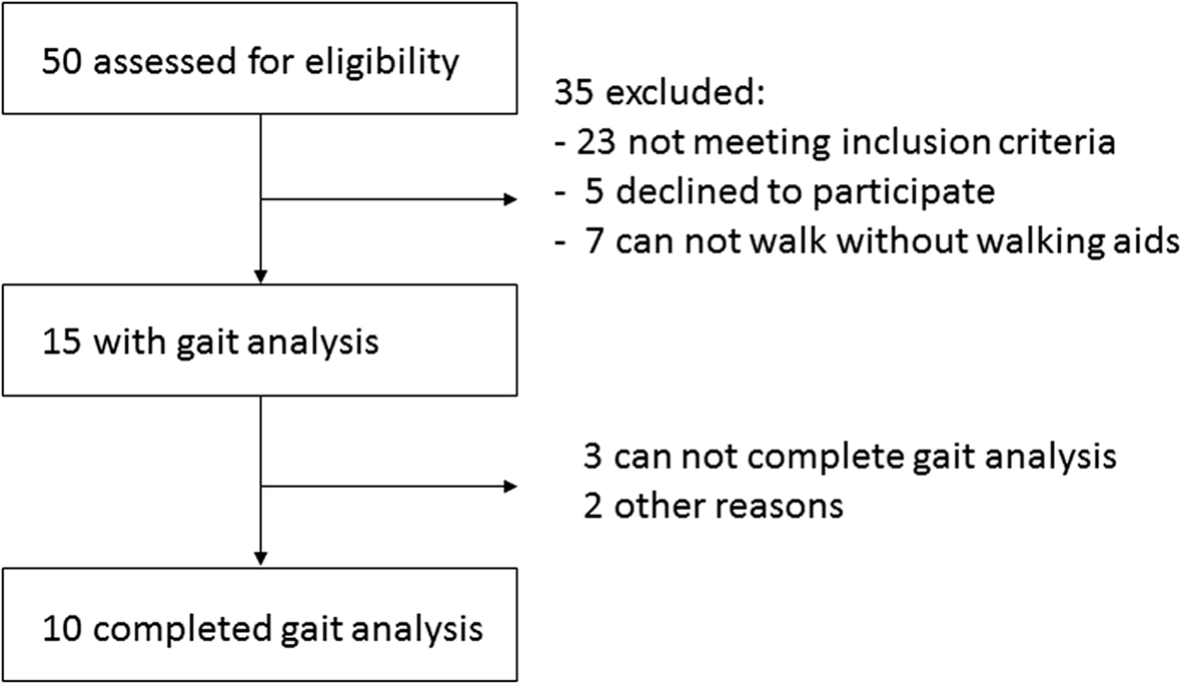
The diagram illustrated the flow of patients’ recruitment

Patient flow through the study is described in Fig. 2. Ten patients, including seven females and three males, who fulfilled our inclusion criteria and ten healthy subjects were enrolled. Five patients had unimalleolar fracture, five patients had bimalleolar fracture. Seven patients received both open reduction and internal fixation and cast immobilization, and three patients received cast immobilization only. Their median score of LEFS was 62 (interquartile range: 26.5). During the 24 months after the fracture, 3 falls were reported by 3 patients.

### Characteristics of demographics for ankle fracture and healthy control participants

The characteristics of the ankle fracture and healthy control groups are listed in Table 1. With the numbers available, no significant difference between the patient and control groups in age, sex, body height, and body weight could be detected.

**Table 1:** Characteristics of demographics for ankle fracture and healthy control participants

Variable	Ankle fracture	Controls	*p*-values*
	Median (IQR)	Median (IQR)	
Age (years)	38.0 (18.0)	39.0 (14.0)	0.85
Sex (men/women)	(3/7)	(3/7)	0.21
Body height (cm)	159.0 (13.0)	157.5 (15.0)	0.25
Body weight (kg)	62.0 (16.8)	56.0 (10.5)	0.13
Time from injury (months)	4.0 (5.0)	NA	NA
Injury side (n)			
Right	8	NA	NA
Left	2	NA	NA

*IQR* interquartile range, *NA* not applicable

* Differences in sex between group were analyzed with the use of Fisher’s exact test. Differences in age, body height, and body weight were analyzed with the use of Mann–Whitney U test

### Comparison of gait parameters between patients with ankle fracture and healthy controls

**Fig. 3:**
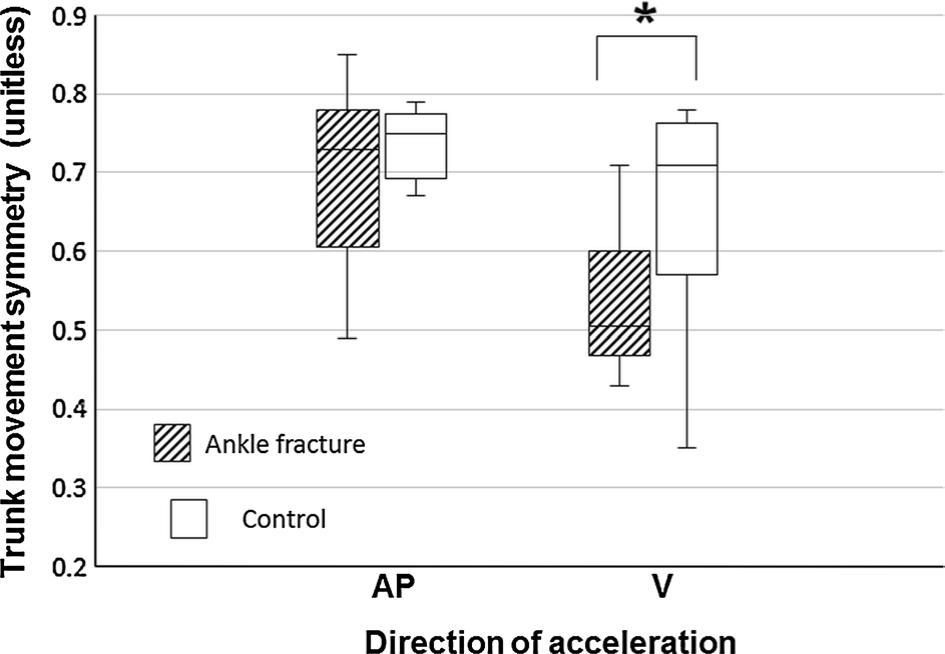
The Box and whisker plot for trunk movement symmetry with and without ankle fractures was illustrated in anteroposterior (AP) and vertical (V) directions, derived from trunk acceleration data with the autocorrelation method. The asterisk indicates *p*-value < 0.05

**Fig. 4:**
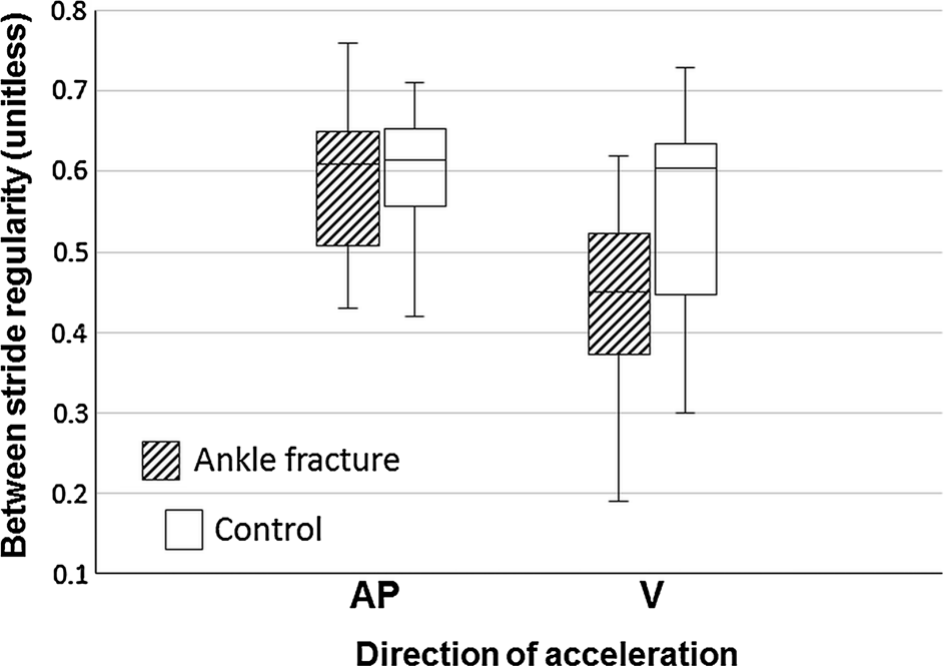
The box and whisker plot for between-stride regularity of trunk movement with and without ankle fractures was illustrated in anteroposterior (AP) and vertical (V) directions, derived from trunk acceleration data with the autocorrelation method

Table 2 reveals the differences of accelerometric profile between ankle fracture and healthy participants. The median step count for analysis were 12.8 steps in ankle fracture group and 9.4 steps in control group. Walking speed (*p *= 0.019), step length (*p *= 0.023), and cadence (*p *= 0.003) were significantly lower in ankle fracture patients than in the control group. Trunk acceleration RMS in AP and VT directions (*p *= 0.001 and *p *= 0.003, respectively) were significantly lower in the ankle fracture group. Symmetry of trunk movement in the VT direction (*p *= 0.019) was significantly lower in the patient group (Fig. 3). Regularity of trunk movement did not significantly differ between groups (Fig. 4). With the numbers available, no significant difference of acceleration RMSR in the ML direction could be detected.

**Table 2:** Comparison of gait parameters between patients with ankle fracture and healthy controls derived from trunk acceleration signals

	Ankle fracture	Control	Absolute difference	*p*-values*
	Median (IQR) (N = 10)	Median (IQR) (N = 10)	Median (95% CI)^a^	
Walking speed (m/s)	0.74 (0.70)	1.28 (0.12)	− 0.53 (− 0.78 to − 0.09)	*0.019*
Step length (cm)	40.5 (19.1)	53.8 (3.0)	− 12.6 (− 20.7 to − 2.4)	*0.023*
Cadence (step/min)	119.0 (29.1)	140.4 (10.1)	− 20.6 (− 58.5 to − 4.7)	*0.003*
AP acceleration RMS (g)	0.09 (0.04)	0.14 (0.05)	− 0.06 (− 0.10 to − 0.01)	*0.001*
Symmetry	0.73 (0.18)	0.75 (0.08)	− 0.03 (− 0.13 to 0.09)	0.631
Stride regularity	0.61 (0.14)	0.62 (0.10)	− 0.01 (− 0.12 to 0.10)	0.853
ML acceleration RMS (g)	0.10 (0.08)	0.13 (0.07)	− 0.01 (− 0.08 to 0.02)	0.165
Acceleration RMSR	0.55 (0.14)	0.44 (0.18)	0.14 (− 0.01 to 0.19)	0.105
VT acceleration RMS (g)	0.11 (0.07)	0.17 (0.05)	− 0.06 (− 0.14 to − 0.02)	*0.003*
Symmetry	0.51 (0.13)	0.71 (0.19)	− 0.19 (− 0.30 to 0.02)	*0.019*
Stride regularity	0.45 (0.15)	0.60 (0.18)	− 0.14 (− 0.27 to 0.05)	0.075

*IQR* interquartile range, *CI* confidence interval, *RMS* root mean square, *RMSR* root mean square ratio, *AP* anterior–posterior, *g* gravity, *ML* medial–lateral, *VT* vertical

* Differences in walking speed, step length, cadence, as well as acceleration RMS, trunk movement symmetry, between-stride regularity between groups were analyzed with the use of Mann–Whitney U test

^a^Absolute differences are given for median values in all gait parameters

Italic values indicate significance of *p * value (*p * < 0.05)

### Correlation analysis between LEFS and gait parameters

Table 3 listed the correlation between the LEFS score and gait parameters. There was a moderately positive rank correlation between step length and LEFS score (r = 0.68; *p *= 0.021), followed by walking speed (r = 0.60; *p* = 0.044); a low-to-little rank correlation was found between other gait parameters and the LEFS score.

**Table 3:** Correlations for gait parameters and Lower Extremity Functional Scale

Variable	LEFS	
	Correlation	*p* value*
Walking speed (m/s)	0.60	*0.044*
Step length (cm)	0.68	*0.021*
Cadence (step/min)	0.50	0.085
AP acceleration RMS (g)	0.57	0.054
Symmetry	0.40	0.142
Stride regularity	0.34	0.183
ML acceleration RMS (g)	0.53	0.072
Acceleration RMSR	0.03	0.466
VT acceleration RMS (g)	0.44	0.120
Symmetry	0.33	0.196
Stride regularity	0.07	0.432

*LEFS* Lower Extremity Functional Scale, *AP* anterior–posterior, *ML* medial–lateral, *VT* vertical, *RMS* root mean square, *RMSR* root mean square ratio

* Correlations between LEFS and gait parameters were analyzed with the use of Spearman’s rank correlation coefficient

Italic values indicate significance of *p * value (*p * < 0.05)

### Correlation analysis between future falls and gait parameters

Table 4 listed the correlation between future falls and gait parameters. There was a high positive rank correlation between acceleration RMSR in ML axis and future falls (r = 0.72; *p* = 0.018) during 24-months follow-up.

**Table 4:** Correlations for gait parameters and future falls after ankle fractures

Variable	Fall	
	Correlation	*p* value*
Walking speed (m/s)	0.27	0.458
Step length (cm)	0.19	0.599
Cadence (step/min)	0.27	0.458
AP acceleration RMS (g)	0.08	0.832
Symmetry	0.08	0.834
Stride regularity	0.27	0.452
ML acceleration RMS (g)	0.31	0.390
Acceleration RMSR	0.72	*0.018*
VT acceleration RMS (g)	0.12	0.752
Symmetry	0.34	0.332
Stride regularity	0.46	0.184

*LEFS* Lower Extremity Functional Scale, *AP* anterior–posterior, *ML* medial–lateral, *VT* vertical, *RMS* root mean square, *RMSR* root mean square ratio

* Correlations between Gait parameters and future falls were analyzed with the use of Spearman’s rank correlation coefficient

Italic values indicate significance of *p * value (*p * < 0.05)

## Discussion

This study showed that symmetry of trunk movement in the VT direction was significantly lower in ankle fracture patients than the control group. Decreased walking speed, step length and cadence were noted in patients following ankle fracture. Furthermore, walking speed and step length were moderately correlated with the LEFS score. Trunk acceleration RMSR in ML axis was highly correlated with future falls.

A high degree of trunk movement symmetry and regularity is a characteristic of a normal gait [13], which means low trunk movement variability between each step or stride. Previous studies had used trunk acceleration data with AC method [33] in patients with transfemoral amputation [36], stroke [37], foot wear prescription [13], and total hip replacement [38]. The AC coefficient of trunk acceleration in the VT axis was found to be a fall predictor in patients with a vertebral or hip fracture [39]. However, few studies concerning trunk movement symmetry and regularity in ankle fracture patients had been found in the current literature.

Our study was novel in that trunk movement symmetry and regularity were evaluated in patients with ankle fracture by an automated infrared-assisted AGA system. Although gait asymmetry of plantar pressure distribution, single-limb support time, and ankle motion following ankle fracture had been investigated by a force plate or 3-dimensional motion analysis system [1, 27], few of them focused on trunk kinematics in early rehabilitation period. Terrier et al. used trunk acceleration data with AC method and found that no between-strides regularity difference was noted between ankle fracture patients and controls [13], which was in accordance with our study. In the present study, between-steps trunk movement symmetry in VT direction was significantly lower in patients with ankle fracture. Reduced plantar flexor moment at the injured ankle joint could interfere with heel contact [1]; weakness of lower trunk muscles such as iliopsoas and gluteal muscles could lead to poor control of vertical acceleration of the centre of gravity during the loading and mid-stance phases of the gait cycle [39]. Decreased range of motion, reduced peak muscle torque, and muscle atrophy of the ankle following immobilization after ankle fracture might be related to displacement of centre of gravity in the sagittal plane, consequently interfering with between-steps trunk movement symmetry in VT axis [40–42]. Rehabilitation strategies should be focused more on the musculoskeletal structures associated with VT direction movement in patients following ankle fractures, such as range of motion exercise or physical modalities to improve ankle motion and strengthening exercise of weak ankle or trunk muscles.

With AC method to analyse trunk accelerometric data, our study did not show trunk movement between-strides irregularity in AP or VT directions in patients following ankle fracture. Unlike those in a previous study [13], our studied patients had a regular gait, which was probably due to different severity of injuries.

Our study revealed that function of physical activities, evaluated by the score of LEFS, was significantly correlated with walking velocity and step length. Conversely, other ankle fracture studies using OMAS did not find high correlation between functional outcomes and measured gait parameters [1, 43]. The reason why LEFS and OMAS had different correlation with gait parameters might be that the two instruments represent different ways to measure outcomes in patients with ankle fractures [44].

Trunk acceleration RMSR in ML axis, the degree of body sway, was found to be positively associated with future falls during 24-months follow-up after ankle fractures. The RMSR in ML axis is found to be associated with walking balance and has a common value at the preferred walking speed of healthy participants that can be used as a threshold for detecting gait abnormalities [32, 45]. It may reflect the chronological change of disease severity effectively in ataxic patients [45]. It was suggested that ankle fracture patents who have high RMSR value should be cautious about risk of future falls.

In the present study, walking speed, step length and cadence were significantly lower in patients with ankle fracture than healthy controls, which was in accordance with a previous study that examined 3-months post-screw removal patients using a portable walkway system [28]. Mor et al. had used a lower limb inertial measurement sensor system to evaluate lower limbs kinematics of post open reduction and internal fixation patients within 6 weeks of partial weight bearing [29]. Although the lower limb system used by Mor et al. could evaluate lower extremity kinematics, it cannot assess basic spatiotemporal gait parameters (walking speed, step length, cadence) at the same time. The automated infrared-assisted, trunk AGA system we used could not only measure basic spatiotemporal gait parameters, but also trunk movement symmetry and regularity simultaneously.

Our study had some limitations. First, our study was adequately powered for the primary outcome (walking speed), but underpowered for several other trunk parameters. It is therefore doubtful that these parameters might have been observed even with larger sample studies. Second, the results obtained in our study represent a subacute stage of rehabilitation following ankle fractures, which may limit its external generalizability. Third, we did not explore the underlying causes of gait deficits. Future research should follow the long-term progress of rehabilitation in gait and trunk movement patterns of patients with ankle fracture.

## Conclusion

Patients following ankle fracture present trunk movement asymmetry in the VT direction as well as altered spatiotemporal gait patterns compared to healthy controls in the early stage of rehabilitation. Proper rehabilitation strategies should be employed to avoid additional injury and optimize functional outcomes. Further studies are needed to follow the long-term progress of rehabilitation outcome in gait and trunk movement patterns of patients with ankle fracture.

## Abbreviations

AC: auto-correlation
AGA: accelerometer-based gait analysis
AP: anterior–posterior
ML: mediolateral
RMS: root mean square
RMSR: root mean square ratio
VT: vertical
